# The tissue micro-array data exchange specification: a web based experience browsing imported data

**DOI:** 10.1186/1472-6947-5-25

**Published:** 2005-08-08

**Authors:** David G Nohle, Barbara A Hackman, Leona W Ayers

**Affiliations:** 1 The Mid-Region AIDS and Cancer Specimen Resource (ACSR), Department of Pathology, The Ohio State University, Columbus, OH USA

## Abstract

**Background:**

The AIDS and Cancer Specimen Resource (ACSR) is an HIV/AIDS tissue bank consortium sponsored by the National Cancer Institute (NCI) Division of Cancer Treatment and Diagnosis (DCTD). The ACSR offers to approved researchers HIV infected biologic samples and uninfected control tissues including tissue cores in micro-arrays (TMA) accompanied by de-identified clinical data. Researchers interested in the type and quality of TMA tissue cores and the associated clinical data need an efficient method for viewing available TMA materials. Because each of the tissue samples within a TMA has separate data including a core tissue digital image and clinical data, an organized, standard approach to producing, navigating and publishing such data is necessary.

The Association for Pathology Informatics (API) extensible mark-up language (XML) TMA data exchange specification (TMA DES) proposed in April 2003 provides a common format for TMA data. Exporting TMA data into the proposed format offers an opportunity to implement the API TMA DES. Using our public *BrowseTMA *tool, we created a web site that organizes and cross references TMA lists, digital "virtual slide" images, TMA DES export data, linked legends and clinical details for researchers.

Microsoft Excel^® ^and Microsoft Word^® ^are used to convert tabular clinical data and produce an XML file in the TMA DES format. The *BrowseTMA *tool contains Extensible Stylesheet Language Transformation (XSLT) scripts that convert XML data into Hyper-Text Mark-up Language (HTML) web pages with hyperlinks automatically added to allow rapid navigation.

**Results:**

Block lists, virtual slide images, legends, clinical details and exports have been placed on the ACSR web site for 14 blocks with 1623 cores of 2.0, 1.0 and 0.6 mm sizes. Our virtual microscope can be used to view and annotate these TMA images. Researchers can readily navigate from TMA block lists to TMA legends and to clinical details for a selected tissue core.

Exports for 11 blocks with 3812 cores from three other institutions were processed with the *BrowseTMA *tool. Fifty common data elements (CDE) from the TMA DES were used and 42 more created for site-specific data. Researchers can download TMA clinical data in the TMA DES format.

**Conclusion:**

Virtual TMAs with clinical data can be viewed on the Internet by interested researchers using the *BrowseTMA *tool. We have organized our approach to producing, sorting, navigating and publishing TMA information to facilitate such review.

We have converted Excel TMA data into TMA DES XML, and imported it and TMA DES XML from another institution into *BrowseTMA *to produce web pages that allow us to browse through the merged data. We proposed enhancements to the TMA DES as a result of this experience.

We implemented improvements to the API TMA DES as a result of using exported data from several institutions. A document type definition was written for the API TMA DES (that optionally includes proposed enhancements). Independent validators can be used to check exports against the DTD (with or without the proposed enhancements). Linking tissue core images to readily navigable clinical data greatly improves the value of the TMA.

## Background

### AIDS and Cancer Specimen Resource

The National Cancer Institute's (NCI) Division of Cancer Treatment and Diagnosis (DCTD) founded the AIDS Malignancy Bank in 1994 to collect various types of specimens from AIDS patients. It was renamed the AIDS and Cancer Specimen Bank and eventually the AIDS and Cancer Specimen Resource (ACSR) to reflect expansion of its role to include seeking out specimens for researchers when needed. Investigators can search a national on-line database [1] to determine what types of specimens are available.

The ACSR provides Tissue Micro-Array (TMA) tissue sections with de-identified clinical data to approved researchers. Researchers interested in the type and quality of TMA core tissues and the associated clinical data can view digital images of hematoxylin & eosin (H&E) stained TMA tissue sections directly on the web. Delay in determining whether it will be fruitful to initiate a request for TMAs is eliminated and visual evaluation of the quality of available material by the researcher is facilitated while preserving the TMA block from unnecessary sectioning [2-4].

### Benefits of using tissue micro-arrays

A tissue micro-array block (TMA) usually is a paraffin recipient block with inserted tiny cylindrical tissue cores taken from donor tissue blocks [5]. A tissue section cut from a TMA block allows large numbers of tissue samples to be tested and stored using less reagent, less effort and less space. Precious tissue is conserved for more research. See the discussion in Berman, et al. [6].

### Clinical data entry

De-identified clinical data is assembled and entered so that it will be available along with the TMA recipient (or mother) block, tissue sections and tissue section virtual images. Excel spreadsheets are used to organize the clinical data and TMA tissue core location assignments. Sequential bank identifiers are assigned by the ACSR database software to replace surgical pathology numbers and hospital identifiers in all communications outside the ACSR. De-identified datasets are used for conversion to TMA DES format once the TMA block has been constructed and entered into database.

### TMA block and slide production

**Figure 1:**
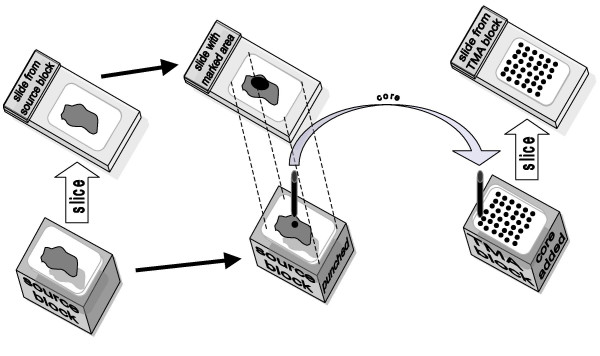
**Producing a TMA block and slide**. A slide from a source block is marked to indicate the area of interest. The marked slide is used to locate the place to punch out a core. The core is extracted from the source block and then inserted into a wax TMA block with cores from other source blocks. TMA slides are then cut from the TMA block.

H&E stained sections of source block tissue on glass or the source embedded tissue paraffin block itself is marked by the pathologist to identify core sites. TMA blocks are then constructed by extracting a tiny (0.6 mm – 2.0 mm diameter) core from all selected source (or donor) tissue blocks and inserting them into a recipient paraffin block (see Figure 1). The most common arrangement is that there is a matrix (rows and columns) of tissue cores in a recipient block and sections are cut from that block.

### Making digitized cut and stained tissue core images

**Figure 2:**
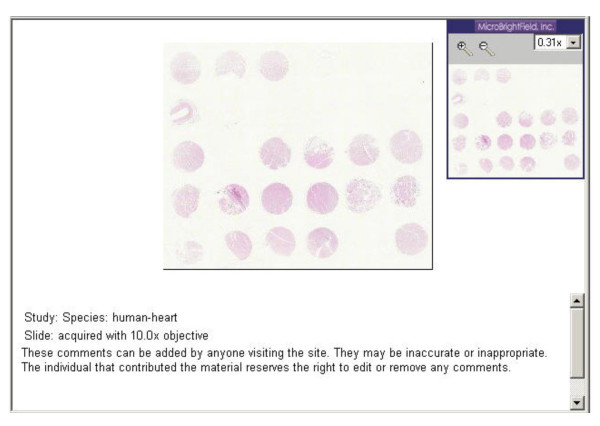
Sample image viewed with Virtual Microscope.

One glass slide with a cut section from a TMA block is typically prepared with H&E stain. A virtual microscope is used to scan and capture a digital image of the entire matrix of cores [7]. This TMA image (see Figure 2) will be made available via the Internet when potential investigators want to see whether slides from this block would be useful in their research. Sending example slides for this purpose is thus avoided.

When a TMA slide is eventually disbursed, the URL of the TMA web page (which links the above mentioned image) and a one page printout of the image are provided to the investigator. These are useful for locating cores in the matrix.

### Data exchange specification

The April 2003 Association for Pathology Informatics (API) TMA data exchange specification (TMA DES) is a proposal for a common XML format for TMA data [8]. A validator (written in Perl) is available with the specification. Exporting TMA data into this format offers an opportunity to apply the specification and validator. Importing and exporting extramural TMA data was used to validate the portability of data using TMA DES.

We used PubMed to access a list of 408 articles related to the TMA DES article [8]. We reviewed the list for implementation and deployment of working examples. We found one other reported implementation [6]. We describe herein our implementation of the TMA DES in a web based experience.

### Cancer Biomedical Informatics Grid

The NCI is relying on the Cancer Biomedical Informatics Grid (caBIG) to expedite research leading to new cancer treatment. Tissue banks will share data on a grid as part of the plan [9]. Investigators must be able to merge results from different sources. TMA tissue section slides from a single TMA block are routinely disbursed to multiple research projects. Standard data formats facilitate the merging of study results and comply with the NCI's emphasis on caGRID conformity.

### Technology

HTML, XML, XSLT and DTD technologies are those principally used; each is introduced in a section below with description of some aspects important to this work. In addition:

• Microsoft Word mail merge main documents are used to convert Excel tab delimited text exports to XML.

• Windows Script Host (WSH aka batch) files [10] are used to invoke routines that process each TMA block.

• A small amount of JScript [11] is used to extend XSLT scripts and to invoke the XSLT parser [12].

• The XSLT Parser MSXML2 4.0 is used to process XML and XSLT files.

### HTML

Most documents on the web are in Hypertext Mark-up Language, HTML. HTML tag delimiters provide instruction for how a document should be displayed [13,14]. For example, the h1 HTML tag delimiter designates a top level heading that a browser will display as such:

   <h1>

      TMA Block: TA00-050

   </h1>

### XML

"XML, the Extensible Mark-up Language, is a W3C-endorsed standard for document mark-up. XML is a metamark-up language for text documents. Data is included in XML documents as strings of text. The data is surrounded by text mark-up that describes the data [15]." The tag delimiters that will be used as mark-up are defined in a particular application and comprise a particular language.

The API specification defines an XML mark-up language for the TMA data representation application so that data from various laboratories can be more readily transmitted, shared or merged [8]. The identifier for a tissue micro-array block, in this case TA00-050, is used as the data text string. The API specified tag delimiter for a block identifier is block_identifier [8]. The marked up data is an XML element:

   <block_identifier>

      TA00-050

   </block_identifier>

When displayed in Microsoft Internet Explorer (as shown in our figures), XML will appear in an indented hierarchy with color-coding to separate mark-up from data.

XML data may be well formed or valid. Well-formed data has matching end tags that are properly nested and follows generic XML rules. It may not necessarily be valid, i.e. follow the rules of a particular language. To assure that data is valid, it should be validated more rigorously using a DTD or checked by other software to ensure that the rules for a particular XML language such as TMA DES are followed.

### XSLT

XSL Transformation (XSLT) is one part of the extensible stylesheet language (XSL). An XSLT document, called an XSLT stylesheet, uses these rules to convert an XML document into another text document, perhaps an HTML document or another XML document.

This bit of XML defines a block containing a block_identifier:

   <block>

      <block_identifier>

         TA00-050

      </block_identifier>

   </block>

This XSLT stylesheet template rule converts an API specified XML block_identifier in the above XML to an HTML top-level heading:

   <xsl:template match="block">

      <h1>

         TMA Block: <xsl:value-of select="block_identifier"/>

      </h1>

   </xsl:template>

Here is the resulting HTML:

   <h1>

      TMA Block: TA00-050

   </h1>

When displayed in a browser the heading will look like this:

   **TMA Block: TA00-050**

"An XSLT processor is a piece of software that reads an XSLT stylesheet, reads an input XML document, and builds an output document by applying the instructions in the stylesheet to the information in the input document. An XSLT processor can be built into a web browser, just as MSXML is in Internet Explorer 6 [15]."

The MSXML processor can be used as a command line processor as well. The processor named MSXML2 version 4.0 is used in *BrowseTMA*.

### DTD

A document type definition (DTD) is used to specify a valid language of XML elements. The DTD can be used with various validators [16,17] to check that XML documents written in this language are valid.

When an external DTD file is referred to and an internal DTD subset is present in an XML file, the two parts collectively are the DTD. The DTD syntax allows definitions to be placed in a file and included into the internal portion as well. The first definition of a given element or entity encountered is used. Internal DTD definitions are encountered before external ones [18].

## Implementation

For each TMA block, a digitized image of a stained TMA section is obtained using the virtual microscope [7].

**Figure 3:**
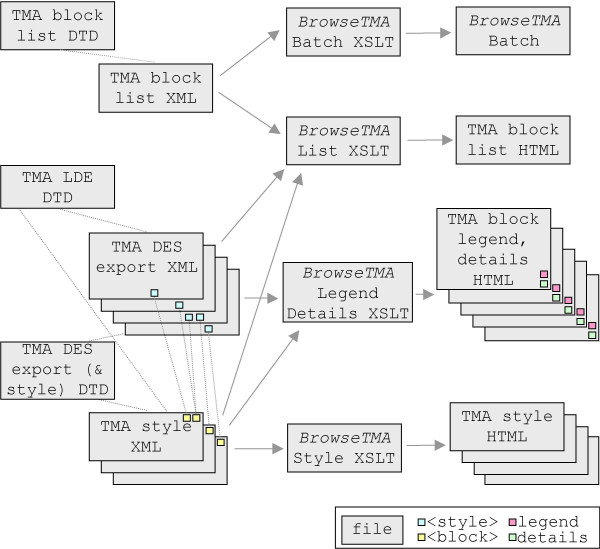
**Implementation**. A DTD governs the allowed data elements in each type (TMA block list, TMA LDE, TMA DES export & style) of XML file. The TMA block list uses *BrowseTMA *to process each listed block file and style. *BrowseTMA *produces a single HTML TMA block list file, a TMA block HTML file for each specified block and an HTML TMA style file for each style used.

The *BrowseTMA *tool was constructed and used to create a web site that organizes and cross references TMA lists, virtual slide images, TMA DES export data, linked legends and clinical details. The TMA data is represented in HTML web pages with automatically added hyperlinks to allow rapid navigation. Figure 3 depicts the modules in *BrowseTMA*.

**Figure 4:**
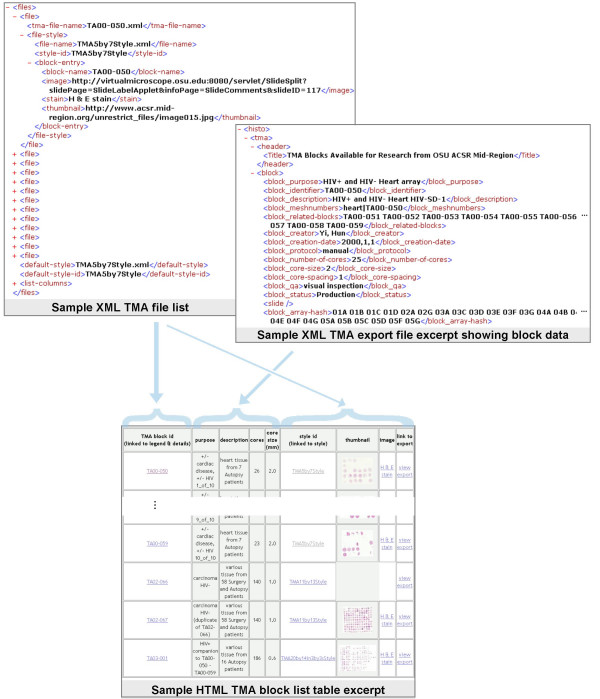
**Producing a block list**. The TMA file list is written in a simple XML language. The language is defined by tmaList.dtd, a document type definition that allows 13 common data elements (ex. tma-file-name, style-id). Referencing this DTD causes validation of the entries indicating which TMA DES export files and blocks to use with which styles. For each TMA file in the TMA file list, *BrowseTMA *processes the specified TMA blocks and displays a row of information about each. In processing a TMA block, *BrowseTMA *uses that block's data from the XML export file to display the export file name, block identifier, purpose and description and the TMA file list entry to display the style file name and links to the style and possibly to an image.

*BrowseTMA *constructs a single block list web page that has a table listing the TMA blocks (linked to each TMA HTML block file and each image) [see fileList.html in Additional file 1]. Figure 4 illustrates this process.

**Figure 5:**
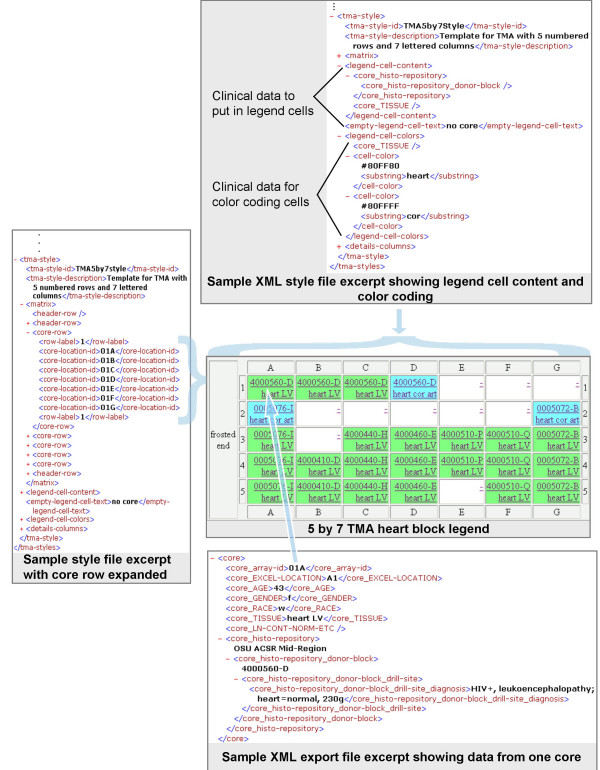
**Producing a legend**. The style file specifies the layout of the array (or matrix) and the legend cell contents and colors using a simple XML language defined in the addStyles conditional section of tmades.dtd. These 26 definitions (ex. core-location-id, row-label, cell-color) allow specification of the TMA layout. They are supplemented by common and locally defined data elements that are nested under the core data element (ex. core_TISSUE, core_histo-repository_donor-block) which may be used to determine the legend cell color and contents and the details columns. Referencing this DTD causes validation of the entries that specify labelling, row and column grouping of cell identifiers, legend cell contents and colors. Cell identifiers (ex. A1, R1C1, or 1.1) are specified in rows with row and column labels interspersed inside the matrix element. The legend-cell-content element contains a list of clinical data items that should be placed in each cell in the HTML legend table. The legend-cell-colors element contains a clinical data item and a list of cell-color elements. The data from the XML export file corresponding to the specified core data item (core_TISSUE is used here) is compared against the substring and exact-match entries inside the cell-color elements and the corresponding color is used for the legend cell in the HTML legend table when a match is found.

**Figure 6:**
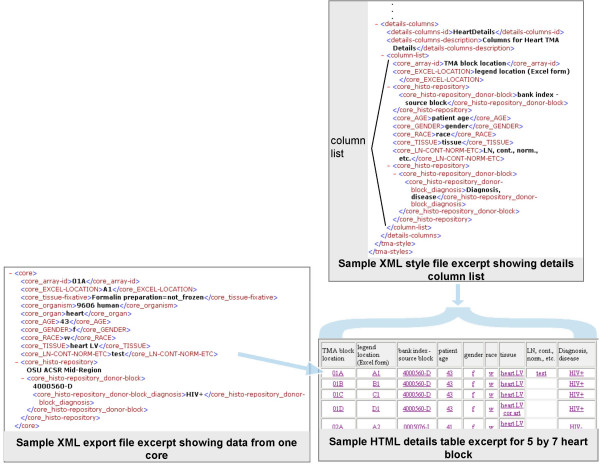
**Producing the clinical details table**. The style file specifies an ordered list of columns and their headings using a simple XML language defined in the addStyles conditional section of tmades.dtd, definitions that allow any data element nested inside the core CDE in the XML export file (ex. core_histo-repository) including locally defined data elements to be used. Activating addStyles causes validation of the entries that specify each details table column. Entries nested inside one or more data elements inside core are allowed. Each core data entry in the XML export file provides one row of cells in the resulting HTML details table.

*BrowseTMA *constructs a web page for each TMA block that contains a details table and a legend table [see TA00-050.html in Additional file 1]. Figures 5 and 6 illustrate these processes. The details table has core specific demographic data copied from the database. The data in each cell is linked to that tissue core's place in the legend table. The legend table, a "roadmap" of the slide, has a link in each cell to the corresponding core's row in the details table.

The various formats involved are discussed below. Descriptions of the activities involved follow.

### DTD for TMA DES language

A DTD governs the allowed common data elements (CDE) in TMA DES export XML files. This DTD is implemented as an external file that is referenced by the TMA DES export XML files and supplemented by internal DTD extensions [18].

CDEs are defined in the external DTD file written at OSU [see tmades.dtd in Additional file 1]. Locally defined data elements (LDE) are in a supplementary DTD file [see myLDEs.dtd in Additional file 1]; this file gets included into the internal DTD section in the TMA DES XML file.

### TMA styles language

Auxiliary XML files are used to specify the TMA styles including fields and color-coding for the legend cells and columns for the details table. Data elements are defined and placed in a conditional section in the external DTD file to govern the style language; this file is referenced as the external DTD in the TMA style XML files. A definition is there activating the style CDEs.

A TMA style file is made for each set of TMA blocks that used a similar pattern. TMA5by7Style is an example [see TMA5by7Style.xml in Additional file 1]. The *BrowseTMA *Style XSLT stylesheet [see BrowseTMAStyle.xsl in Additional file 1] produces an HTML TMA style web page file for each style [see TMA5by7Style.html in Additional file 1].

### TMA DES XML data

Data for a variety of TMA blocks from several institutions is used. OSU produced the first exports in the TMA DES format [2]; the Cooperative Prostate Cancer Tissue Resource (CPCTR) has since produced exports in XML [6,19]. Data from Brigham and Women's Hospital (BWH) and the Tissue Array Research Program (TARP) [20] has been converted to XML at OSU. The *BrowseTMA *Legend Details XSLT stylesheet [see BrowseTMALegendDetails.xsl in Additional file 1] produces a TMA block HTML file for each block.

To produce the TMA DES file for each block, three sets of information are created for each block:

• A text file is entered containing description, tissue, # patients, #cores, Core Size (mm), Autopsy/Surgical/Mixed and ID columns and a row for each block. A block list Word main merge file is merged with the text file to produce the block portion of the TMA DES data for each block.

• The table containing the clinical data in that block's Excel file is exported from the Excel program to a tab delimited text file. It is used for two operations:

     A hash Word main merge file is merged with the text file to produce the block_array-hash element contents for that block.

     A core Word main merge file is merged with the text file to produce the core elements for that block.

These three sets of information are merged by hand for each block.

### TMA block list language

A DTD governs the allowed data elements in a TMA block list XML file [see tmaList.dtd in Additional file 1]. A block list file is used in two ways. The *BrowseTMA *List XSLT stylesheet [see BrowseTMAList.xsl in Additional file 1] uses a TMA block list XML file (as well as export and style files) to produce single HTML TMA block list file [see fileList.html in Additional file 1]. The *BrowseTMA *Make WSH batch file [see BrowseTMAMakeBatch.bat in Additional file 1] invokes the *BrowseTMA *Batch XSLT stylesheet [see BrowseTMABatch.xsl in Additional file 1] which uses a TMA block list XML file [see fileList.xml in Additional file 1] to produce a single batch file [see BrowseTMA.bat in Additional file 1].

### Producing TMA exports

Mail merge was used to produce the bulk of an export from an Excel file [2]. Berman, et al. have discussed how to produce an export from an Excel table using a Perl script and hand editing [8].

### Validating TMA exports with a DTD

We use both cited validation sites to validate a sample TMA block, TA00-050 [see TA00-050.xml in Additional file 1]. No errors are present; warnings may indicate that certain entities are redefined. This is expected as placeholders are defined in the common (external) DTD file and the placeholders are overridden by the local (internal) portion of the DTD.

A combination of XSLT and JScript is used to make a reorder program, BrowseTMAReorder, which accepts a TMA DES XML file and creates a version with certain data elements (mainly the identifiers) first within their parent data element. If no identifier is present one is created using automatic numbering. This allows validation with an improved version of the DTD [18].

### TMA styles

There are a variety of ways to arrange tissue cores within a TMA. The number of rows and columns and the size of the cores, space between cores and between groupings of cores vary. Likewise, there are various ways to designate cells, rows and columns. It is common for a TMA producer to assemble multiple TMA blocks that follow the same pattern.

The way that the matrix of cores is laid out is referred to herein as a TMA style. A simple XML language is used to specify the block layout and the data content and appearance of the legend and details HTML tables. Once specified, a TMA style can be reused for similar TMA blocks. A style file may contain multiple TMA styles.

The TMA style uses a simple XML language specified in the style extensions in tmades.dtd which supplement 33 of the CDEs (those nested under core) from the TMA DES and 31 LDEs from the local DTD with 26 style data elements. Activating these definitions (in an internal DTD subset by redefining the entity addStyles as INCLUDE) enables validation of the entries that specify the style (layout), legend (labelling, spacing, rows and implied columns of core location, cell contents and colors) and details (clinical data column order and labels) tables.

### Specifying matrix layout and legend

A legend is a map of the location of cores in a TMA block, slide or image. In our HTML legend tables, each cell that represents a core in the legend has a hyperlink to that core's row in the HTML details table.

In the matrix element in a TMA style, core location (and matrix cell) identifiers (ex. A1, R1C1, or 1.1) are specified in rows with row and column labels interspersed.

In a TMA style, a few clinical data core attributes are specified so as to appear in HTML legend cells. The legend-cell-content element contains a list of clinical data items that should be placed in each cell in the HTML legend table. Any data elements nested under the core element, whether defined locally or in the TMA DES, are allowed.

In the legend-cell-color element in a TMA style, a single clinical data core attribute may be identified so as to govern the background color of HTML legend cells. Any data element nested under the core element, whether defined locally or in the TMA DES is allowed. Multiple cell-color entries may follow. Inside each cell-color entry, a color (ex. Blue or #8080FF) is followed by either a substring or exact-match element containing a string. The data from the XML export file corresponding to the specified core data item is compared against the substring and exact-match string entries inside the cell-color elements and when the first match is found, the corresponding color is used as the background for the legend cell in the HTML legend table.

### Specifying the details table contents

Each row in the details table has clinical data associated with a core. Each cell in the row has a hyperlink to the legend cell for this core.

The style file specifies an ordered list of columns (and their headings). Each column list entry could be any data element used inside the core CDE in the XML export file (ex. core_histo-repository) including any of the locally defined LDEs. Referencing this DTD causes validation of the entries that specify which clinical data item to include in each HTML details table column. Entries nested inside one or more CDEs inside core are allowed. Each core entry in the XML export file provides one row of cells in the resulting HTML details table.

### Specifying the block list

A TMA publisher should determine which TMA XML export files are to be used and which blocks and styles are to be used together. This information (block and style names and filenames, etc.) should be manually typed in a text file named fileList.xml [see Additional file 1] in the file list format specified by tmaList.dtd [see Additional file 1].

### Producing an HTML block list table

For each TMA file in the TMA file list, *BrowseTMA *processes the specified TMA blocks (or all when none specified) and displays a row of information about each indicated TMA block. Refer to Figure 4. In processing each TMA block, *BrowseTMA *uses that block's data from the XML export file to display the export file name, block identifier, purpose and description.

### Producing an HTML legend table

The style file specifies the layout of the array (or matrix) and the legend cell contents and colors [see tmades.dtd in Additional file 1]. Activating the style definitions in the DTD allows validation of the entries that specify labelling, row and column grouping of cell identifiers, legend cell contents and colors. For each TMA file, *BrowseTMA *processes the specified TMA blocks (or all when none specified) and creates an HTML file containing a legend table (followed by a clinical details table as described below) for each indicated TMA block. Refer to Figure 5. In processing each TMA block, *BrowseTMA *uses that block's data from the XML export file to display the export file name, block identifier, purpose and description as the header and specified attributes in each cell in the legend table.

### Producing an HTML clinical details table

The style file specifies the details table columns. Activating the style definitions in the DTD allows validation of the entries that specify the core attributes, headings and order. Refer to Figure 6. *BrowseTMA *processes the specified TMA block and displays a row of information about each core in the block. In processing each core, *BrowseTMA *uses that block's data from the XML export file to display the fields specified for each details column.

## Results and discussion

Block lists, virtual slide images, legends, clinical details and exports are on the ACSR web site for 14 blocks with 1623 cores of 2.0, 1.0 and 0.6 mm sizes. Exports for 11 blocks with 3812 cores from three other institutions are processed with the *BrowseTMA *tool. Researchers can readily navigate from TMA block lists to TMA legends and to clinical details for a selected tissue core. Our virtual microscope can be used to view and annotate linked TMA images. Researchers can download TMA clinical data in the TMA DES format.

Fifty common data elements (CDE) from the 80 defined by the API TMA DES are used. 42 local data elements (LDE) are defined to store data that cannot be mapped to the API TMA DES definitions. This information can help indicate whether additional CDEs should be added to the specification. Table 1 shows the LDEs with indication of which institutions they were used for. For example, as race and tissue elements are needed for data from three institutions, adding CDEs for these might be appropriate. Detailed comments about converting TMA data to the TMA DES format are provided [see Additional file 2].

**Table 1:** LDE with use by institution

*ParentCDE*	*LDE*	*OSU*	*CPCTR*	*BWH*	*TARP*
block	block_NUMBER-OF-PATIENTS	x			
	block_AUTOPSY-SURGICAL-MIXED	x			
core	core_HIV-STATUS	x			
	core_EXCEL-LOCATION	x		x	
	core_AGE	x			x
	Year_of_Birth		x		
	Year_of_Diagnosis		x		
	Year_of_Prostatectomy		x		
	core_GENDER	x			x
	core_RACE	x			x
	Race		x		
	core_TISSUE	x		x	x
	core_LN-CONT-NORM-ETC	x			
	core_COMMENTS	x		x	
	core_COORDINATES			x	
	IMS_Case_Identifier		x		
	Location_Code		x		
	Is_Residual_Carcinoma_Present		x		
	Most_Prominent_Histologic_Type		x		
	Gleason_Primary_Grade		x		
	Gleason_Secondary_Grade		x		
	Gleason_Sum_Score		x		
	Number_of_Nodes_Examined		x		
	Number_of_Nodes_Positive		x		
	Distant_Mets__1_at_Time_of_Diagn		x		
	pT_Stage		x		
	pN_Stage		x		
	pM_Stage		x		
	Vital_Status		x		
	Year_of_PSA_Recurrence		x		
	PSA_Recurrence_Status		x		
	Recurrence_Free_Year		x		
	core_OTHER-INFO-NOTES				x
	core_COUNT				x
	core_BLOCK-SIZE				x
	core_STATUS				x
	core_PROTOCOL				x
	core_FIXATION				x
	core_ORGAN				x
	core_IHC				x
	core_ORGANISM				x
	core_GRADE				x

The data from the CPCTR is modified to make it compatible with the *BrowseTMA *capabilities. Identifier LDEs were changed to CDEs to avoid additional programming that is necessary when required information (such as a block, core or slide identifier) is stored in locally defined data elements minimizing the benefit of using a common format. Detailed comments about modifying CPCTR data are provided [see Additional file 2].

Six recommendations are prompted by difficulties experienced exporting, importing and using data in the TMA DES format. Several relate to confusion about 'sections'. The data structure section of the specification [8] describes dividing any TMA DES XML file into 4 sections. This is misleading because when there are multiple blocks, the core CDEs are not all together like the chapters of a book. The structure of such XML files should be understood to be a hierarchy or tree. It would be clearer to say that data elements are grouped into 4 categories: header, block, slide and core. A data element of the respective type is the root of a sub-tree that contains data elements about that respective header, block, slide or core. The idea of 'sections' breaks down because each core and slide element must be inside some block element (i.e. within that block section). Detailed recommendations are provided [see Additional file 2].

## Conclusion

An organized approach to producing, sorting, navigating and publishing TMA information using the *BrowseTMA *tool has been put in place. Virtual TMA sections with clinical data from the ACSR can be evaluated on the Internet by interested researchers. Linking images to readily navigable clinical data facilitates reasearcher evaluation of the TMA.

A DTD has been written and applied to the API TMA DES specification and is in routine use. Using a DTD (optionally reflecting our proposed enhancements) can provide stronger validation. The *BrowseTMA *tool can be used to merge XML exports from various sources and to construct an integrated website. The *BrowseTMA *tool and DTD are available publicly to assist TMA DES users in reviewing and validating the XML exports they have constructed.

### Next steps

We are considering enhancing *BrowseTMA *to:

• use more slide and block information from exports as defaults to define the block list including multiple slides per block (displayed in the block list), slide image locations, multiple blocks per file,

• allow selection of block list fields as in details,

• navigate to the specific core image (not just the image for the whole block),

• handle namespaces,

• provide details table sorting options,

• improve element attribute handling,

• allow result column addition and editing,

• define and publish allowed data element values and

• produce printer friendly versions of all pages (smaller font, minimum width, no links, legend separated from details).

We plan to rework the style elements to fit inside the histo/tma hierarchy.

We hope to interact with other interested parties about exporting and merging TMA data and improvements to the TMA DES.

## Availability and requirements

Example TMA HTML and XML data files and all source code for the *BrowseTMA *tool and related software are available at the public ACSR Mid-Region web site  Personal computers running Microsoft Windows 98, 2000, NT and XP have been used with Internet Explorer 6.0, Word 2002, and FrontPage 2002 in this work.

## List of abbreviations used

ACSR – AIDS and Cancer Specimen Resource

AIDS – acquired immunodeficiency syndrome

AMB – AIDS Malignancy Bank

API – Association for Pathology Informatics

caBIG – cancer Biomedical Informatics Grid

CDE – common data elements

DCTD – Division of Cancer Treatment and Diagnosis

DES – data exchange specification

DTD – document type definition

H&E – hematoxylin & eosin

HIV – human immunodeficiency virus

HTML – hypertext markup language

IRB – institutional review board

LDE – locally defined data elements

NCI – National Cancer Institute

OSU – The Ohio State University

TMA – tissue micro-array

TMA DES – tissue micro-array data exchange specification

URI – universal resource identifier

W3C – World Wide Web Consortium

XML – extensible mark-up language

## Competing interests

The author(s) declare that they have no competing interests.

## Authors' contributions

DGN developed the Microsoft Mail Merge scripts, Excel formulas, API DES DTD, JScript programs, WSH batch scripts and *BrowseTMA *XSLT scripts and wrote the first draft of the manuscript. BAH supervised the construction of the TMA blocks and slides, provided clinical data and scanned slides with the virtual microscope. LWA was responsible for the TMA design including case selection, block selection, core focus selection and core quality assurance. All authors reviewed and commented on successive drafts of the manuscript and versions of the software and have approved the final manuscript.

## Pre-publication history

The pre-publication history for this paper can be accessed here:



## Supplementary Material

Additional file 1 This .zip file contains the following 16 files which may be extracted using standard unzip software: **Sample TMA DES export: TA00-050.xml **This TMA block contains 35 cores in a 5 by 7 array. This XML file can be viewed with Internet Explorer or another browser or editor. **Sample TMA file list: fileList.xml **Entries for all but one TMA block are commented out to reduce the number of files needed to try the software while illustrating more substantive use. This XML file can be viewed with any text editor or browser. **Sample TMA style file: TMA5by7Style.xml **The sample TMA block uses the TMA style specified in this TMA style file. TMA5by7Style specifies a 5 row by 7 column array. In legend tables based on this style: • Column labels are repeated at top and bottom. • The label end of the block/slide is at left. • Row labels are repeated at left and right. • The bank ID and organ are shown in each legend cell, which is color-coded based on diagnosis. In details tables based on this style, common and locally defined data elements containing column labels are ordered so that top-to-bottom data elements can be map to left-to-right output table columns. This XML file can be viewed with Internet Explorer or another browser or editor. **TMA DES DTD: tmades.dtd **This DTD defines the CDEs (tags) and allowed structure of API TMA DES exports. Also present are elements to define the allowed structure of TMA styles in TMA style files. It can be viewed with any text editor. **TMA file list DTD: tmaList.dtd **This DTD defines the CDEs (tags) and allowed structure of a TMA file list. It can be viewed with any text editor. **TMA local data element file DTD: myLDEs.dtd **This file defines the LDEs (tags) and their allowed structure. It can be viewed with any text editor. **BrowseTMA XSLT script: BrowseTMABatch.xsl **This XSLT file produces a single batch file, BrowseTMA.bat, which contains commands to run the BrowseTMAList.xsl XSLT script once and the BrowseTMAStyle.xsl and BrowseTMALegend.xsl XSLT scripts for each TMA block specified in fileList.xml. **BrowseTMA WSH batch file: BrowseTMA.bat **This WSH batch file runs the BrowseTMAList.xsl XSLT script once and the BrowseTMAStyle.xsl and BrowseTMALegend.xsl XSLT scripts for each TMA block specified in fileList.xml. Standard output is redirected to the appropriate HTML file. **BrowseTMA XSLT script: BrowseTMAStyle.xsl **This XSLT file produces a single TMA style HTML file hyperlinked to the TMA block list and from the blocks that use this style. **BrowseTMA XSLT script: BrowseTMAList.xsl **This XSLT file produces a single TMA file list HTML file hyperlinked to each TMA block and style HTML file referenced in it. **BrowseTMA XSLT script: BrowseTMALegendDetails.xsl **This XSLT file produces a single TMA block HTML file containing a legend and details table hyperlinked to each other. **Sample TMA legend and details: TA00-050.html **This displays the legend and details tables for the sample TMA block represented in the sample TMA DES block export in TA00-050.xml. This HTML file can be viewed with Internet Explorer or other browser. **Sample TMA file table: fileList.html **This displays the files, blocks and styles given in the sample TMA file list, fileList.xml. This HTML file can be viewed with Internet Explorer or other browser. **Sample TMA style table: TMA5by7Style.html **This displays the TMA5by7Style style specified in TMA5by7Style.html. This HTML file can be viewed with Internet Explorer or other browser. **XSLT test JScript: xsltTest.js **This JScript program runs the Microsoft XSLT Parser, MSXML2 4.0, on a passed XML file and XSLT file. **BrowseTMA Make WSH batch file: BrowseTMAMakeBatch.bat **This WSH batch file runs the BrowseTMABatch.xsl XSLT script once and outputs the BrowseTMA.bat file. Click here for file

Additional file 2 This HTML document contains detailed comments and recommendations regarding use of the TMA DES. It can be viewed with Internet Explorer or other browser. Click here for file
