# Supplementary material for: The tissue micro-array data exchange specification: a web based experience browsing imported data
Source: BMC Med Inform Decis Mak. 2005 Aug 8;5:25. doi: 10.1186/1472-6947-5-25 (PMC1208890; doi:10.1186/1472-6947-5-25)
Supplement: Additional file 1 — This .zip file contains the following 16 files which may be extracted using standard unzip software: Sample TMA DES export: TA00-050.xml This TMA block contains 35 cores in a 5 by 7 array. This XML file can be viewed with Internet Explorer or another browser or editor. Sample TMA file list: fileList.xml Entries for all but one TMA block are commented out to reduce the number of files needed to try the software while illustrating more substantive use. This XML file can be viewed with any text editor or browser. Sample TMA style file: TMA5by7Style.xml The sample TMA block uses the TMA style specified in this TMA style file. TMA5by7Style specifies a 5 row by 7 column array. In legend tables based on this style: • Column labels are repeated at top and bottom. • The label end of the block/slide is at left. • Row labels are repeated at left and right. • The bank ID and organ are shown in each legend cell, which is color-coded based on diagnosis. In details tables based on this style, common and locally defined data elements containing column labels are ordered so that top-to-bottom data elements can be map to left-to-right output table columns. This XML file can be viewed with Internet Explorer or another browser or editor. TMA DES DTD: tmades.dtd This DTD defines the CDEs (tags) and allowed structure of API TMA DES exports. Also present are elements to define the allowed structure of TMA styles in TMA style files. It can be viewed with any text editor. TMA file list DTD: tmaList.dtd This DTD defines the CDEs (tags) and allowed structure of a TMA file list. It can be viewed with any text editor. TMA local data element file DTD: myLDEs.dtd This file defines the LDEs (tags) and their allowed structure. It can be viewed with any text editor. BrowseTMA XSLT script: BrowseTMABatch.xsl This XSLT file produces a single batch file, BrowseTMA.bat, which contains commands to run the BrowseTMAList.xsl XSLT script once and the BrowseTMAStyle.xsl and BrowseTMALegend.xsl XSLT scripts f [file 1472-6947-5-25-S1.zip › TA00-050.htm]

TA00-050 in TA00-050.xml
using TMA5by7Style in TMA5by7Style.xml


## Linked Legend & Details for TA00-050

heart tissue from 7 Autopsy patients

+/- cardiac disease, +/- HIV 1\_of\_10

### Using Style TMA5by7Style

Template for TMA with 5 numbered rows and 7 lettered columns

### TA00-050 Legend

|
|  |
| frosted end |  | A | B | C | D | E | F | G |  |
| 1 | 4000560-D heart LV | 4000560-D heart LV | 4000560-D heart LV | 4000560-D heart cor art | - | - | - | 1 |
| 2 | 0005076-I heart cor art | - | - | - | - | - | 0005072-B heart cor art | 2 |
| 3 | 0005076-I heart LV | - | 4000440-H heart LV | 4000460-E heart LV | 4000510-P heart LV | 4000510-Q heart LV | 0005072-B heart LV | 3 |
| 4 | 0005076-I heart LV | 4000410-D heart LV | 4000440-H heart LV | 4000460-E heart LV | 4000510-P heart LV | 4000510-Q heart LV | 0005072-B heart LV | 4 |
| 5 | 0005076-I heart LV | 4000410-D heart LV | 4000440-H heart LV | 4000460-E heart LV | - | 4000510-Q heart LV | 0005072-B heart LV | 5 |
|  | A | B | C | D | E | F | G |  |

#### Go to Mid-Region ACSR Tissue Micro-Array List

### TA00-050 Details

|  |  |  |  |  |  |  |  |
| --- | --- | --- | --- | --- | --- | --- | --- |
| TMA block location | bank index - source block | patient age | gender | race | tissue | LN, cont., norm., etc. | Diagnosis, disease |
| 01A | 4000560-D | 43 | f | w | heart LV |  | HIV+, leukoencephalopathy; heart=normal, 230g |
| 01B | 4000560-D | 43 | f | w | heart LV |  | HIV+, leukoencephalopathy; heart=normal, 230g |
| 01C | 4000560-D | 43 | f | w | heart LV |  | HIV+, leukoencephalopathy; heart=normal, 230g |
| 01D | 4000560-D | 43 | f | w | heart cor art |  | HIV+, leukoencephalopathy; heart=normal, 230g |
| 01E | - |  |  |  |  |  |  |
| 01F | - |  |  |  |  |  |  |
| 01G | - |  |  |  |  |  |  |
| 02A | 0005076-I | 41 | f | w | heart cor art |  | HIV-, pulmonary embolus; heart=normal, 350g |
| 02B | - |  |  |  |  |  |  |
| 02C | - |  |  |  |  |  |  |
| 02D | - |  |  |  |  |  |  |
| 02E | - |  |  |  |  |  |  |
| 02F | - |  |  |  |  |  |  |
| 02G | 0005072-B | 26 | m | w | heart cor art |  | HIV-, Hodgkin's lymphoma; heart=interstitial fibrosis, 510g |
| 03A | 0005076-I | 41 | f | w | heart LV |  | HIV-, pulmonary embolus; heart=normal, 350g |
| 03B | - |  |  |  |  |  |  |
| 03C | 4000440-H | 43 | m | w | heart LV |  | HIV+, pneumonia; heart=normal, 370g |
| 03D | 4000460-E | 33 | m | w | heart LV |  | HIV+, lymphoma; heart=lymphoma, 440g |
| 03E | 4000510-P | 35 | m | w | heart LV |  | HIV+, pneumonia; heart=interstitial fibrosis, 370g |
| 03F | 4000510-Q | 35 | m | w | heart LV |  | HIV+, pneumonia; heart=interstitial fibrosis, 370g |
| 03G | 0005072-B | 26 | m | w | heart LV |  | HIV-, Hodgkin's lymphoma; heart=interstitial fibrosis, 510g |
| 04A | 0005076-I | 41 | f | w | heart LV |  | HIV-, pulmonary embolus; heart=normal, 350g |
| 04B | 4000410-D | 40 | m | b | heart LV |  | HIV+, lung carcinoma; heart=normal (RV=fibrosis), 350g |
| 04C | 4000440-H | 43 | m | w | heart LV |  | HIV+, pneumonia; heart=normal, 370g |
| 04D | 4000460-E | 33 | m | w | heart LV |  | HIV+, lymphoma; heart=lymphoma, 440g |
| 04E | 4000510-P | 35 | m | w | heart LV |  | HIV+, pneumonia; heart=interstitial fibrosis, 370g |
| 04F | 4000510-Q | 35 | m | w | heart LV |  | HIV+, pneumonia; heart=interstitial fibrosis, 370g |
| 04G | 0005072-B | 26 | m | w | heart LV |  | HIV-, Hodgkin's lymphoma; heart=interstitial fibrosis, 510g |
| 05A | 0005076-I | 41 | f | w | heart LV |  | HIV-, pulmonary embolus; heart=normal, 350g |
| 05B | 4000410-D | 40 | m | b | heart LV |  | HIV+, lung carcinoma; heart=normal (RV=fibrosis), 350g |
| 05C | 4000440-H | 43 | m | w | heart LV |  | HIV+, pneumonia; heart=normal, 370g |
| 05D | 4000460-E | 33 | m | w | heart LV |  | HIV+, lymphoma; heart=lymphoma, 440g |
| 05E | - |  |  |  |  |  |  |
| 05F | 4000510-Q | 35 | m | w | heart LV |  | HIV+, pneumonia; heart=interstitial fibrosis, 370g |
| 05G | 0005072-B | 26 | m | w | heart LV |  | HIV-, Hodgkin's lymphoma; heart=interstitial fibrosis, 510g |

#### Go to Mid-Region ACSR Tissue Micro-Array List
