# Supplementary material for: The tissue micro-array data exchange specification: a web based experience browsing imported data
Source: BMC Med Inform Decis Mak. 2005 Aug 8;5:25. doi: 10.1186/1472-6947-5-25 (PMC1208890; doi:10.1186/1472-6947-5-25)
Supplement: Additional file 2 — This HTML document contains detailed comments and recommendations regarding use of the TMA DES. It can be viewed with Internet Explorer or other browser. [file 1472-6947-5-25-S2.htm]

Comments & recomendations from using the TMADES


# Comments and recommendations from using the tissue micro-array data exchange specification (TMA DES)

## Mapping data to common data elements (CDE)

While
determining how to convert TMA data to the TMA DES format, we collected the
following comments.

 

1.     
Often a block contains multiple
tissue cores from the same patient and sometimes from the same source block. A patient or source-block element would
eliminate repetition of patient data in every core that
uses it.

 

2.     
It is necessary to remove �&� from all data items (we
changed them to �+� or to �&amp;�).

 

3.     
Our criterion for using a CDE
vs. contriving a locally defined element (LDE) was that it faithfully labelled
the data. As the allowed values are not defined in the TMA DES or the external
DTD made for it, TMA blocks from different institutions used the same CDE
differently. While all LDEs may be candidates for
addition to the specification, those used for multiple institutions carry more
weight.

 

4.     
Some CDEs
require numbers to be in millimeters yet have
examples with the units in the data. It is unclear whether mm should appear in the block\_core-size data and block\_core-spacing.

 

5.     
The block\_core-size CDE assumes that there is a uniform size. Smaller control cores are
sometimes used. It is unclear whether it be routine to
leave these cores out of the data so that the assumption is valid.

 

6.     
Virtual microscopes allow a
single digital image to be produced for the entire TMA section but there is no
CDE defined to associate the address with the slide CDE. A slide\_image
CDE defined to be similar to the core\_image CDE,
together with similar CDEs to those nested under the core\_image, would remedy this.

## Reformatting TMA exports for *BrowseTMA*

The data from the CPCTR was modified to
make it compatible with the *BrowseTMA* capabilities. The following comments resulted:

 

1.     
We removed the namespace
declarations from the histo
CDE as the *BrowseTMA*
tool does not currently handle namespaces.

 

� <histo xmlns="http://65.222.228.150/jjb/tma\_cde.htm"

���
xmlns:cpctr=

����� "http://www.pathology.pitt.edu/pdf/cpctr/...

���
xmlns:dc="http://dublincore.org">

 

2.     
We also removed the namespace
prefixes (dc: and cpctr:) from the elements
that used them.

 

3.     
The CPCTRTMA.xml file seemed to
contain information about four blocks (named cpctr-01-01,
cpctr-01-02, cpctr-02-01 and cpctr-02-02 judging from the block\_related-blocks CDE)
with up to 300 cores in each. There was only one block CDE in the file but there were four LDEs
of the form block\_*x*\_*y*. As we wish to handle CDEs so that we can reliably identify block designations,
we replaced these with four block CDEs and added a block\_identifier CDE in each with the cpctr-0*x*-0*y* name. We believe that the
specification should mandate that information about only one block is within a block CDE and that a block\_identifier CDE is present in each.

 

4.     
The CPCTRTMA.xml file only had one
set of block\_\* information (CDEs that start with block\_); we
copied it into each block and adjusted the block\_related-blocks accordingly to list the other three blocks in each case. The block\_related-blocks CDE
has been used at OSU to list other blocks from the same study with no cores in
common (where multiple blocks were required to hold enough heart tissue cores
to accomplish the investigation). Perhaps the definition in the specification
should not preclude such use.

 

5.     
In the CPCTRTMA.xml file, the block\_array\_hash CDE contained a
lengthy free text description of the core placements. We think that the
specification should mandate that this CDE contain a list of the core\_array-ids that can possibly
be used in the block. Perhaps the descriptive text should be added to the block\_description.
We did not alter the block\_array-hash as it did not disrupt the processing we were doing.

 

6.     
In the CPCTRTMA.xml file, each block
only contained one core CDE (a total of four
in the file). There were many record CDEs; each seemed to be about one or two cores from the
same source/donor block as designated in the array\_locations LDE. Once again, to handle CDEs so that
we can reliably identify core
designations, we replaced each record LDE
with a core CDE and replaced each array\_location LDE
with a core\_array-id CDE. Where two locations were specified, we copied the entire core
section and edited each core\_array-id to contain a single row and column. We also abbreviated the format
to r*X*c*Y* to save screen width and
reduce clutter. We think the specification should mandate that information
about only one core be within a core CDE and
a core\_array-id CDE should be present in each. An alternative would be to allow
multiple core\_array-id entries within a single core entry when all other attributes within the core were
the same for those core-array-id entries.

## Using TMA DES exports

Here are the
recommendations prompted by difficulties experienced exporting, importing and
using data in this format:

 

1.     
The data structure section of the specification
[6] describes dividing any TMA DES XML file into 4 sections. This is misleading
because the core (and slide) CDEs are not necessarily all together in the file (if there
are multiple blocks) like the chapter of a book. The structure of such XML
files should be understood to be a hierarchy or tree, like a computer file
system. It would be better to say that data elements could be conceptually
grouped into 4 categories:
header, block, slide and core. A data element of the respective type is the
root of a sub-tree that contains data elements about that respective header,
block, slide or core. The idea of sections breaks down because each core and slide element must be
nested inside some block element (i.e. within
that block �section�).

 

It may not be
clear that a section does not correspond to a single header, block, slide or core data element but many.
For example, data about all cores in a TMA block should not be lumped together
under a single core data element; there
should typically be a core data element (with
data about that core nested inside it) for each core in that TMA block. See our
revised version of �The data
structure� section below.

 

**The data structure**

Every TMA file is an XML file to be understood as a hierarchy or
tree (like a computer file system). Almost all of the data can be grouped into
4 categories:

 

1. A header category, with data elements that provide basic
information about the file (creator, date created, etc.). The header elements
are taken directly from the Dublin Core, a set of specification elements used
in libraries throughout the world to index electronic information files http://dublincore.org. These elements are
nested in a single header data element.

 

2. A block category, with data elements that describe the TMA
block (how many cores, how large are cores, how are the cores arrayed in the
block, etc.). Many block data elements, one for each TMA block, may be present.
Each contains data elements about a particular TMA block.

 

3. A slide category, with data elements that describe the slides
prepared from the TMA block (how are the slides stored, how are they
identified, etc). Many slide data elements, one for each TMA slide, may be
present and will be nested within the block data element for the TMA block that
they were cut from. Each slide data element will contain data elements that
describe one particular slide.

 

4. A core category with data elements that describe each of the
cores in the TMA block (what case did the core come from, what block from the
case was used to make the core, what drill-site in the block was used, what was
the diagnosis of the drill-site, what clinical history is associated with the
core, what demographic information is associated with the patient from whom the
core was taken, etc.). This category is by far the largest category, with
well-annotated data for every core in the TMA block. Many core data elements,
one for each TMA core, may be present and will be nested within the block data
element for the TMA block that they were inserted into. Each core data element
will contain data elements that describe one particular core.

 

2.     
Rule 3 of the Semantic rules for the TMA data exchange
specification section [6] should likewise avoid using the term section.
It should also state that any data present should apply to the single block, slide or core data element in which it is nested. Here is a revised version:

 

Every TMA file must have tma, header,
block, slide, and core data elements. Each block, slide or core data element
will contain only information that describes the object represented by data
element.

 

3.     
The header CDE Maximum Occurrence
is given as Unlimited. The filename CDE is described as the name of the document file. As this is the
name of the TMA dataset that contains the information nested inside a tma CDE, there could
not be more than one per dataset. Note that when multiple datasets are concatenated,
each dataset can result in another tma CDE which
contains its own header CDE. Any other arrangement which allows multiple header
CDEs and multiple block CDEs
nested in the same tma CDE will cause ambiguity about
the relationship between each block CDE and header CDE without providing any
useful benefit. The header Maximum
Occurrence should be changed to one.

 

4.     
The Maximum Occurrence is given as Unlimited on most CDEs. We interpret
the Maximum Occurrence field as specifying the number allowed within a single
parent element (otherwise, since there is no limit on the tma datasets placed within a histo element in a file, all other CDEs
would be Unlimited). Allowing multiple of most of these CDEs
can only introduce ambiguity, i.e. what does it mean to have multiple dates,
types, IDs or formats within a parent CDE? We propose that all CDEs except the following be changed to have Maximum
Occurrence of One: histo, Creator, Publisher, Contributor, filename, tma,
block, block\_creator, core, core\_clinical-info,
core\_histo-repository\_specimen-diagnosis, core\_histo-repository\_donor-block\_drill-site\_diagnosis, core\_results, and core\_image.

 

5.     
Although there is some utility
in simply encoding data into XML (self-describing data, data sharing, etc.), a
common specification is intended to accomplish more than getting data into (any
kind of) XML. The utility of the TMA DES certainly rests on the understanding
that there is a firm hierarchy relating blocks, cores and slides. Without a
regular way to discern the relationships between the block, core and slide
data, very little standard functionality can be implemented. For example, a
legend cannot be produced.

 

The main two
mechanisms available to specify relationships in TMA DES XML files seem to be:

 

- hierarchical nesting - used to indicate that the sections in slides represented by
  the slide CDEs inside a block CDE were cut from that block, core CDEs inside a block CDE represent cores that were
  inserted in that block, child data elements (even if locally defined)
  inside a parent data element are about the parent, etc. While we may
  assume that these relationships are meant, they should be explicitly
  stated in the specification.

 

- identifiers as pointers - used in the core\_array-id and block\_array-hash CDEs to locate cores in the array. The specification
  should state that the core\_array-id should contain only one location
  identifier for the core
  that this element is nested in. The block\_array-hash
  should not be allowed to contain descriptive text but instead should
  contain an ordered row-by-row list of the location designations in the
  array. We would further suggest that the specification specify a row CDE to be used inside and contain the left to right ordered
  list of core\_array-id identifiers that so that the matrix layout is fully specified.

 

6.     
Elements to define the style of
a TMA could be added to the specification. The element definitions used in *BrowseTMA* provide a starting point for this.
